# Supplementary material for: Transcriptome Analysis of Maize Ear Leaves Treated with Long-Term Straw Return plus Nitrogen Fertilizer under the Wheat–Maize Rotation System
Source: Plants (Basel). 2023 Nov 16;12(22):3868. doi: 10.3390/plants12223868 (PMC10674774; doi:10.3390/plants12223868)
Supplement: Supplementary file 1 [file plants-12-03868-s001.zip › plants-2671105-supplementary.pdf]

Supplementary Materials: The following supporting information can be downloaded at: www.mdpi.com/xxx/s1, Supplementary Table S1: Summary of the RNA-seq clean data; Supplementary Table S2: Summary of mapping results; Supplementary Table S3: KEGG enrichment pathways of the DEGs in three pair-wise comparisons with *P*-value < 0.05; Supplementary Table S4: Fertilization rates in the experiment field per year (kg ha^{-1}); Supplementary Table S5: Primers used for qRT-PCR validation; Supplementary Figure S1: Changes of photosynthetic parameters responsive to various fertilizer treatments; Supplementary Figure S2: Volcano plots of DEGs in SRvsCK, NvsCK and SRNvsCK comparisons; Supplementary Figure S3: GO barplots of DEGs in SRvsCK, NvsCK and SRNvsCK comparisons. CC, cell component; BP, biological process; MF, molecular function.

Table S1. Summary of the RNA-seq clean data.

Samples	Total reads	GC content (%)	Q20 (%)	Q30 (%)	Mean Quality Score	Total bases (G)
CK_1_clean_R1	47202746	55.48	98.98	96.53	36.5	6.8569
CK_1_clean_R2	47202746	55.96	98.85	95.56	36.36	6.9911
CK_2_clean_R1	46683398	55.73	99.17	96.99	36.57	6.8439
CK_2_clean_R2	46683398	55.76	99.33	97.06	36.46	6.9105
CK_3_clean_R1	32261285	55.12	99.24	97.15	36.6	4.7436
CK_3_clean_R2	32261285	55.98	98.64	94.91	36.26	4.7698
SR_1_clean_R1	35820990	54.4	99.07	96.7	36.53	5.2458
SR_1_clean_R2	35820990	54.81	97.21	92.96	36.21	5.0793
SR_2_clean_R1	38439945	54.37	99.11	96.81	36.54	5.6294
SR_2_clean_R2	38439945	55	97.24	93.35	36.27	5.4465
SR_3_clean_R1	42700145	54.69	99.03	96.66	36.52	6.2331
SR_3_clean_R2	42700145	55.03	97.3	93.37	36.27	6.0623
N_1_clean_R1	41440011	55.95	99.15	96.97	36.57	6.0741
N_1_clean_R2	41440011	56.59	98.9	95.77	36.4	6.1378
N_2_clean_R1	36789597	55.81	99.16	96.98	36.57	5.3908
N_2_clean_R2	36789597	56.52	98.95	95.95	36.43	5.4511
N_3_clean_R1	51770564	56.42	99.09	96.88	36.56	7.5524
N_3_clean_R2	51770564	57.03	98.96	95.98	36.43	7.6765
SRN_1_clean_R1	37482289	54.05	99.1	96.78	36.54	5.4951
SRN_1_clean_R2	37482289	54.44	97.43	93.79	36.34	5.323
SRN_2_clean_R1	35659272	54.87	99.03	96.68	36.53	5.2078
SRN_2_clean_R2	35659272	55.08	97.24	93.31	36.27	5.0556
SRN_3_clean_R1	41194730	49	99.42	97.68	36.49	5.873
SRN_3_clean_R2	41194730	51.54	98.85	95.93	36.44	6.0428
**Total**	**974889944**					**142.0922**

Table S2. Summary of mapping results.

Sample	Clean reads	Mapped reads	non-unique	unique	Read-1	Read-2	Reads map to '+'	Reads map to '-'	Mapped rate	Concordant pair alignment rate
CK_1	94405492	71201915	29116949	42084966	20987181	21097785	21141294	20943672	75.42%	71.43%
CK_2	93366796	70049954	18192168	51857786	25942811	25914975	26039585	25818201	75.03%	70.76%
CK_3	64522570	51060622	10829590	40231032	20068246	20162786	20213681	20017351	79.14%	74.21%
SR_2	76879890	61086315	7368859	53717456	26915677	26801779	26998084	26719372	79.46%	73.55%
SR_3	85400290	66691878	16119708	50572170	25351703	25220467	25430032	25142138	78.09%	72.91%
N_1	82880022	64577231	18037756	46539475	23280989	23258486	23373108	23166367	77.92%	73.41%
N_2	73579194	58056031	15667670	42388361	21194615	21193746	21296282	21092079	78.90%	74.38%
N_3	103541128	78833107	27023698	51809409	25919026	25890383	26021118	25788291	76.14%	72.12%
SRN_1	74964578	60790745	8527835	52262910	26171016	26091894	26250855	26012055	81.09%	75.46%
SRN_2	71318544	55545165	13503201	42041964	21088533	20953431	21129183	20912781	77.88%	73.22%
SRN_3	82389460	61610969	17431385	44179584	21872531	22307053	22249926	21929658	74.78%	68.51%
**Total**	**176794952**	**132812884**		**86264550**						

Table S3. KEGG enrichment pathways of the DEGs in three pair-wise comparisons with *P*-value < 0.05.

Comparisons	KEGG enrichment pathway	Gene Ratio	DEGs number
SRvsCK	Carbon metabolism	0.079	26
	Starch and sucrose metabolism	0.061	20
	Circadian rhythm - plant	0.052	17
	Cysteine and methionine metabolism	0.039	13
	Carbon fixation in photosynthetic organisms	0.03	10
	Arginine and proline metabolism	0.024	8
	Galactose metabolism	0.024	8
	Pentose phosphate pathway	0.024	8
	Nitrogen metabolism	0.021	7
	Carotenoid biosynthesis	0.021	7
	Sphingolipid metabolism	0.021	7
	Nicotinate and nicotinamide metabolism	0.012	4
	Other glycan degradation	0.012	4
	Brassinosteroid biosynthesis	0.009	3
	Glucosinolate biosynthesis	0.006	2
NvsCK	Biosynthesis of amino acids	0.101	23
	Carbon metabolism	0.079	18
	Starch and sucrose metabolism	0.075	17
	Carbon fixation in photosynthetic organisms	0.053	12
	Glycerolipid metabolism	0.053	12
	Amino sugar and nucleotide sugar metabolism	0.048	11
	Glycerophospholipid metabolism	0.044	10
	Alanine, aspartate and glutamate metabolism	0.031	7
	Nitrogen metabolism	0.026	6
	ABC transporters	0.018	4
	Linoleic acid metabolism	0.0132	3
	Benzoxazinoid biosynthesis	0.013	3
SRNvsCK	Plant hormone signal transduction	0.088	44
	Phenylpropanoid biosynthesis	0.064	32
	Starch and sucrose metabolism	0.056	28
	MAPK signaling pathway - plant	0.056	28
	Circadian rhythm - plant	0.036	18
	Ribosome biogenesis in eukaryotes	0.034	17
	Galactose metabolism	0.024	12

	Carotenoid biosynthesis	0.02	10
	Steroid biosynthesis	0.018	9
	Fatty acid elongation	0.018	9
	Nitrogen metabolism	0.016	8
	Phenylalanine metabolism	0.016	8
	Benzoxazinoid biosynthesis	0.012	6
	ABC transporters	0.012	6
	Nicotinate and nicotinamide metabolism	0.01	5

Table S4. Fertilization rates in the experiment field per year (kg ha^{-1}).

**Treatments**	**Straw return**	**N fertilizer**
CK	0	0
SR	14,301	0
N	0	276
SRN	14,301	276

Note: In SR treatment, both wheat straws (6694 kg ha^{-1}) and maize straws (7607 kg ha^{-1}) were crushed and return the field. Urea was used as an N fertilizer.

Table S5. Primers used for qRT-PCR validation.

**Gene**	**Primer sequence**	
	**Forward (5'-3')**	**Reverse (5'-3')**
GRMZM2G173387	CCTCCTGAAATCACCGACAAGTCC	TGGCGTGAGAACACCGTCCC
GRMZM2G154090	GCGCAGAACCAGGACAAGAGCAAG	CACGAGGAAGGTGAGGATAAAGCC
GRMZM2G045473	ATCGGTTTCCTGTTCACGTTCCTGG	TCACTCAAATCTGTCGGCACGGTCT
GRMZM2G326707	GCGCAAGAGCGTGTACGGGATGA	CCGAGCCAGAAGCGGAAGAAGCA
GRMZM2G455124	GGGCTCACCTTTGGCATCGTCC	CATGTACTTGATCCCCGTCTCCGTCTT
GRMZM5G878558	ACCAGCCCGACGACGACACG	TCTCCCCACGCCGTACTCCCAC
GRMZM2G102959	GGCTGCATATCTGCCTGCCCTCT	GCTGCTGTCCCTGTGCTGTGGTG
AGPL2	GGTTCCAGGGCACAGCAGAC	AAGCTCCATGTAATCCATACGATA
GRMZM2G025833	TTCCTGTCGTGGTACTCGCAGATGC	GCTGCCGTAATGCCAGTGGATG
GRMZM2G108133	GCTTCTGCATGAGATGGGTGTCG	TCGTCCTGAAGAGCCTGTGGG
GRMZM2G008226	ACCGGATTTCCTGATGTGCG	GCTGGGCTTCTGACCGACTG
GRMZM2G078472	AACCACCCATCACGAGTTCCATTTC	CCATCTTCACGCCCAGCGACTT
Rpi2	GGGTGTTGGTGCTCGCTTTGTC	GACCTCAGCCTGGCGTGGAAT
GRMZM5G871471	TTTGTGCTGCTGCTTATGTTGC	AAGATGAATCCACCGTGAACTG
VR	AGGTGGCGGCGAAGGGGATCA	CGGGCGCTATGTCTGTCGTCTCG
ZmActin1	GTCCATGAGGCCACGTACAA	CCGGACCAGTTTCGTCATA

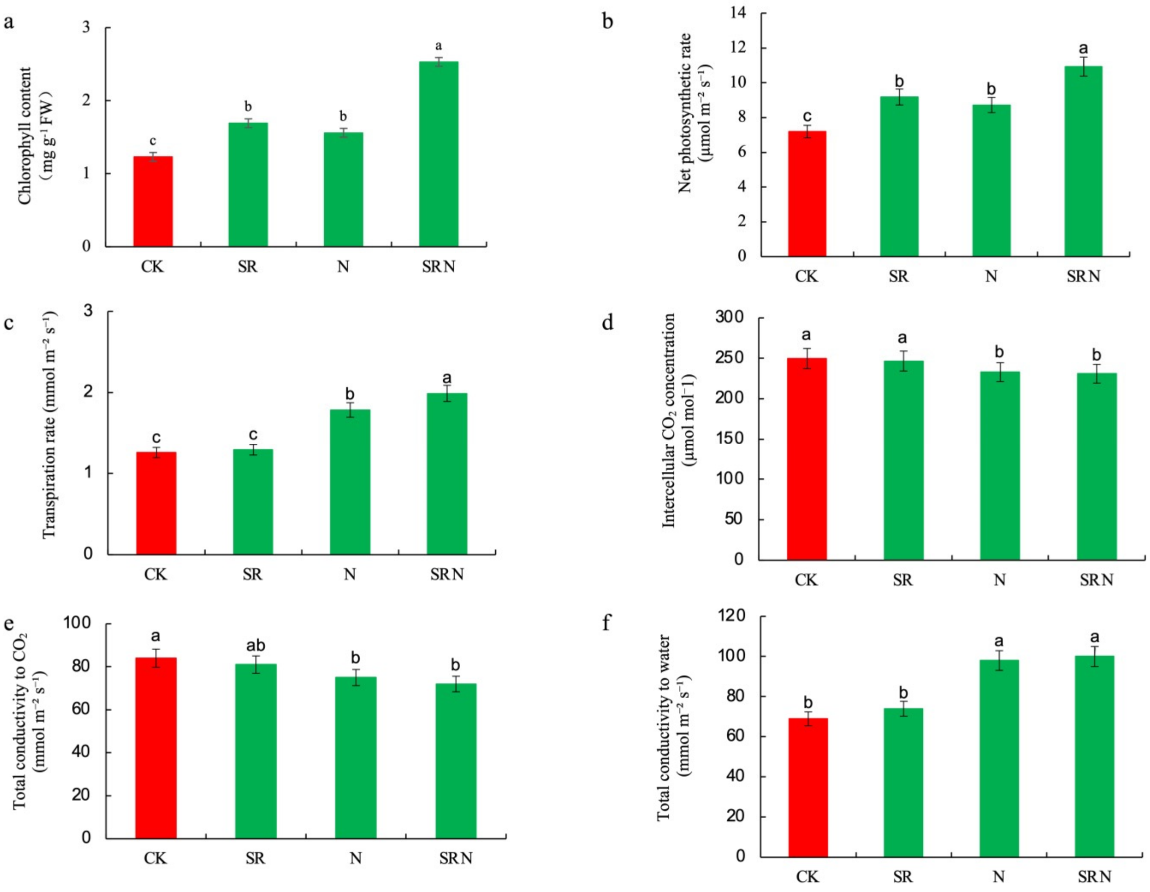

Figure S1. Changes of photosynthetic parameters responsive to various fertilizer treatments. (a), chlorophyll content; (b), net photosynthetic rate; (c), transpiration rate; (d), intercellular CO_2 concentration; (e), total conductivity to CO_2; (f), total conductivity to H_2O_2. Data shown are means ± SE, n = 3. Different letters indicate statistically significant differences between treatments at *P*-value < 0.05.

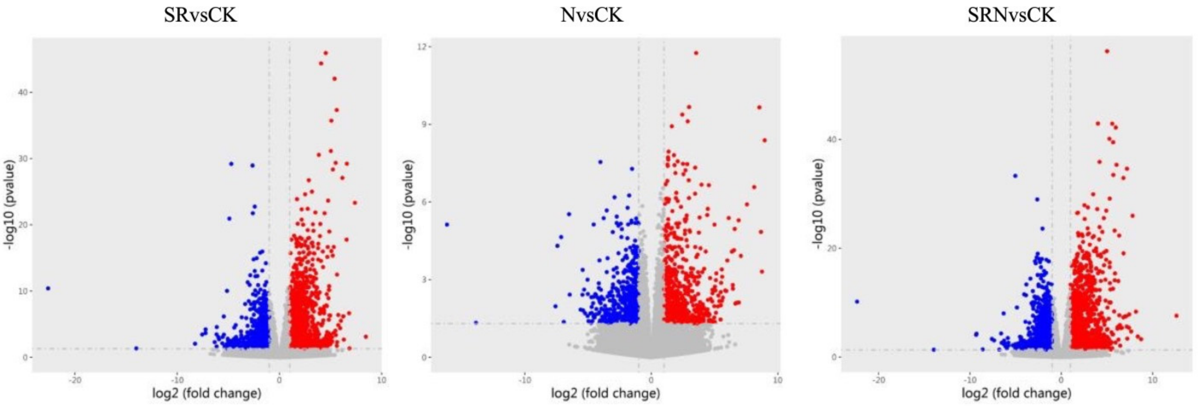

Figure S2. Volcano plots of DEGs in SRvsCK, NvsCK and SRNvsCK comparisons.

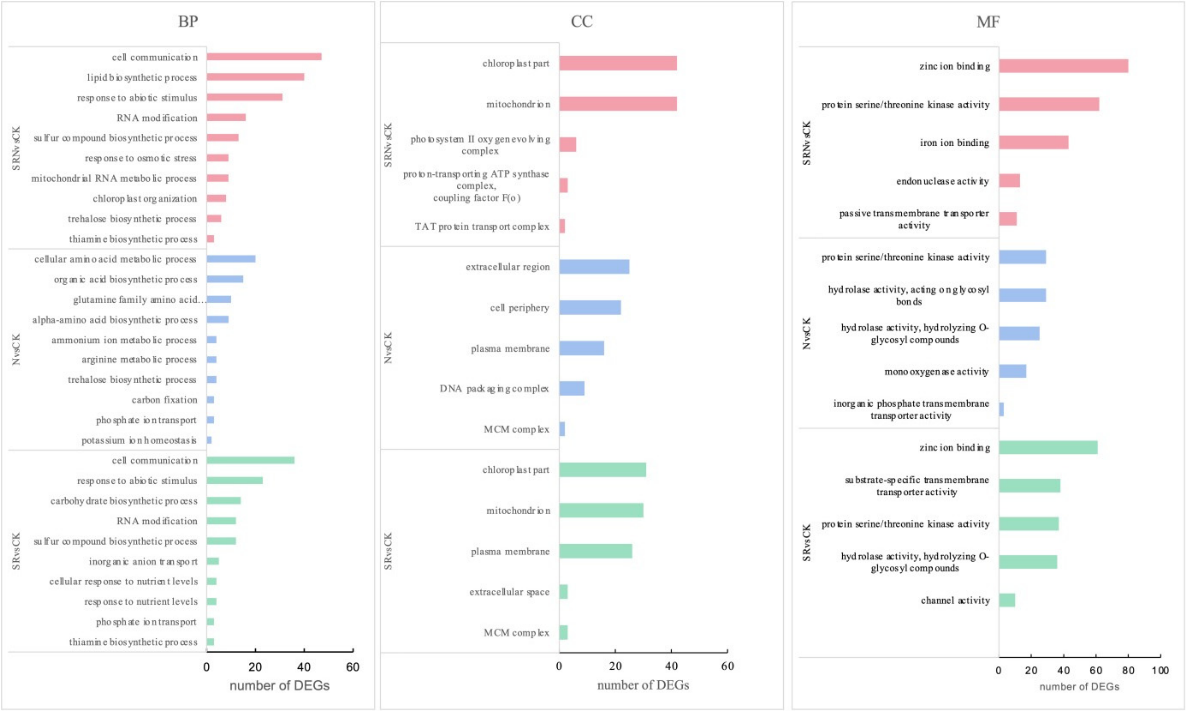

Figure S3. GO barplots of DEGs in in SRvsCK, NvsCK and SRNvsCK comparisons. CC, cell component; BP, biological process; MF, molecular function.
